# Meta-Analysis and Advancement of Brucellosis Vaccinology

**DOI:** 10.1371/journal.pone.0166582

**Published:** 2016-11-15

**Authors:** Tatiane F. Carvalho, João Paulo A. Haddad, Tatiane A. Paixão, Renato L. Santos

**Affiliations:** 1 Departamento de Clínica e Cirurgia Veterinárias, Escola de Veterinária, Universidade Federal de Minas Gerais, Belo Horizonte, MG, Brazil; 2 Departamento de Medicina Veterinária Preventiva, Escola de Veterinária, Universidade Federal de Minas Gerais, Belo Horizonte, MG, Brazil; 3 Departamento de Patologia Geral, Instituto de Ciências Biológicas, Universidade Federal de Minas Gerais, Belo Horizonte, MG, Brazil; East Carolina University Brody School of Medicine, UNITED STATES

## Abstract

**Background/Objectives:**

In spite of all the research effort for developing new vaccines against brucellosis, it remains unclear whether these new vaccine technologies will in fact become widely used. The goal of this study was to perform a meta-analysis to identify parameters that influence vaccine efficacy as well as a descriptive analysis on how the field of *Brucella* vaccinology is advancing concerning type of vaccine, improvement of protection on animal models over time, and factors that may affect protection in the mouse model.

**Methods:**

A total of 117 publications that met the criteria were selected for inclusion in this study, with a total of 782 individual experiments analyzed.

**Results:**

Attenuated (n = 221), inactivated (n = 66) and mutant (n = 102) vaccines provided median protection index above 2, whereas subunit (n = 287), DNA (n = 68), and vectored (n = 38) vaccines provided protection indexes lower than 2. When all categories of experimental vaccines are analyzed together, the trend line clearly demonstrates that there was no improvement of the protection indexes over the past 30 years, with a low negative and non significant linear coefficient. A meta-regression model was developed including all vaccine categories (attenuated, DNA, inactivated, mutant, subunit, and vectored) considering the protection index as a dependent variable and the other parameters (mouse strain, route of vaccination, number of vaccinations, use of adjuvant, challenge *Brucella* species) as independent variables. Some of these variables influenced the expected protection index of experimental vaccines against *Brucella* spp. in the mouse model.

**Conclusion:**

In spite of the large number of publication over the past 30 years, our results indicate that there is not clear trend to improve the protective potential of these experimental vaccines.

## Introduction

Brucellosis is a zoonotic bacterial disease that accounts for approximately half a million new cases of human infections annually [1]. The disease is caused by different *Brucella* species, which are facultative intracellular Gram negative bacteria that belong to the α-2 Proteobacteriacea family [2,3]. Human patients with brucellosis develop nonspecific symptoms including undulating fever, and the disease may progress to endocarditis, arthritis, osteomyelitis, among other less common clinical manifestations [4]. In cattle, brucellosis is characterized by abortion and infertility [5–7]. Therefore, bovine brucellosis results in very significant economic losses [8,9].

Animal brucellosis control and prevention is largely based on vaccination. Therefore, over the past decades there has been an intensive research effort for developing safer and more efficacious vaccines against brucellosis [3,10–12]. Animal vaccination against brucellosis is based mostly on live attenuated vaccines [12], including *Brucella abortus* S19, *Brucella abortus* RB51, and *Brucella melitensis* Rev.1 [3,11,13], whereas *Brucella abortus* S19 is often considered a gold standard for vaccine development [14]. However, these live attenuated vaccine strains have some significant disadvantages including pathogenic potential for humans, induction of abortion in animals, shedding in the milk, and interference with serologic tests in the case of smooth LPS strains [3,15]. Furthermore, these traditional vaccine strains have their use restricted to ruminants, whereas pigs, camels, or wild life animals are not covered.

Traditionally, live attenuated vaccines have a much broader use and efficacy than inactivated vaccine formulations [12,16]. During the past few years, there have been an increasing number of studies on alternative approaches for immunization against brucellosis, including recombinant subunit vaccines using surface or intracellular proteins of *Brucella* spp. [17–20]. Several *Brucella* proteins have been used as immunogens for experimental subunit vaccine formulations, including outer membrane proteins, namely Omp16, Omp19, Omp31, Omp28, and Omp25 [21–24], ribosomal protein L7/L12 [17,25], Cu-Zn superoxide dismutase [26], a cytoplasmic protein p39 [27], lumazine synthase BLS [28], among others. In addition, experimental DNA vaccines [28,29] as well as vectored vaccines using deliver vectors such as *Salmonella enterica* serotype Typhimurium [30], *Escherichia coli* [31], *Yersinia enterocolitica* [32], *Lactococcus lactis* [33], and the influenza virus [34] have been increasingly studied. Overexpression of *Brucella* antigens in attenuated vaccine strains have also been experimentally evaluated [35]. However, up to date these new approaches have not resulted in the generation of commercially available vaccines.

Due to the limitations of experimental procedures involving the natural hosts, since it is expensive and time-consuming, the mouse has been largely used as an experimental model for vaccine development against brucellosis [15]. The mouse model is suitable for studying pathogenesis, host immune response, and vaccine protection [36,37]. However, experimental protocols for assessing vaccine efficacy using this animal model are not standardized, which generates results that are often not quite reproducible [38]. Balb/c is the most commonly used mouse strain, although other strains have also been used for vaccine experiments, namely CD1, C57BL/6, OF1, 129/Sv, Swiss, and, mixed/outbred [16]. Vaccine efficacy is assessed based on experimental challenge with a pathogenic wild type *Brucella* strain after immunization, and quantification of wild type bacteria in target organs, particularly the spleen [39].

In spite of all the research effort for developing new vaccines against brucellosis, it remains unclear whether these new vaccine technologies will in fact become widely used tools for preventing brucellosis. Therefore, the goal of this study was to perform a meta-analysis to identify parameters that influence vaccine efficacy as well as a descriptive analysis on how the field of *Brucella* vaccinology is advancing in regard to type of vaccine, improvement of protection on animal models over time, and factors that may affect protection in the mouse model.

## Material and Methods

### Data source

Data were retrieved from publications indexed in PubMed up to February 15^th^ 2016, using the following combinations of terms: (i) “*Brucella*” and “vaccine”; (ii) “*Brucella*” and “vaccine” and “mice”; or (iii) “*Brucella*” and “vaccine” and “mice” and “challenge”. The list of publications were then manually disambiguated. Only papers using the mouse model were included in this study. Importantly, a criterion for inclusion was that the paper must indicate the protective index or provide original data that allowed us to calculate the index. By definition, protective index refers to the difference in the log of colony forming unit (CFU) numbers in the spleen of naive and vaccinated mice. Only papers published in English were included in this study. In addition, papers with insufficient data–i.e. absence of indication of number of mice per group, absence of CFU values with their standard deviation, and absence of non vaccinated controls–were not included in this study.

### Data retrieval

This study was performed according to the Preferred Reporting Items for Systematic Reviews and Meta-Analyses criteria (PRISMA) as detailed in S1 Table. Data were obtained from each individual experimental group in a given publication. These data were grouped according to the category of experimental vaccine being tested, including: (i) live attenuated strains, (ii) DNA vaccines; (iii) inactivated vaccines; (iv) mutant attenuated strains; (v) subunit vaccines; and (vi) vectored vaccines. Parameters extracted from each individual experiment and considered for analysis included: publication year, vaccine species (in the case of live vaccines), protection index, mouse strain, variables related to vaccination (route, dose, number of injections, and adjuvant), variables related to the challenge (challenge *Brucella* species and strain, route, and interval in days between challenge and sampling), vector species was considered in the case of vectored vaccines.

A linear regression analysis was performed considering the year of publication and protection index, for all experiments or grouped according to the category of vaccine. In addition, the influence of each parameter (category of vaccine, mouse strain, route of vaccination and challenge, number of vaccinations, adjuvant, challenge species, and interval between challenge and euthanasia) on the protective index.

### Data transformation and meta-regression analysis

Arbitrary values were attributed to qualitative data. For instance, values from 0 to 5, being “0” for attenuated vaccines; “1” for DNA vaccines; “2” for inactivated vaccines; “3” for mutant vaccine strains; “4” for subunit vaccines; and “5” for vectored vaccines. Similarly, values were attributed to mouse strains, routes of vaccination and challenge, use of adjuvant, *Brucella* spp. species used for challenge, and number of vaccinations, applying the value zero to the reference and integral crescent values to the other categories. The interval between challenge and euthanasia was analyzed as linear quantitative data.

The coefficient of variation, standard error, and confidence intervals were calculated for each experiment included in this study.

### Statistical analysis

The analysis was conducted initially a random effects meta-analysis estimation with a heterogeneity test. If the heterogeneity test is significant (p-value lower than 0.05), and probable would be significant because there are different types of study with different types of vaccines, it is necessary to work using a meta-regression in order to verify which factor has positive or negative effect over the protective index.

The conduction of the meta-regression would use first two independent variables, one always the type of vaccine with the objective of control the effect of the second independent variable. In this “controlled univariate meta-regression” will conduct checking the association of independent variables such as mouse strain, vaccination route, number of vaccinations, use of adjuvant, *Brucella* species used for challenge, route of challenge, interval between challenge and euthanasia; and the dependent variable Protective Index. The independent variables with over-all p-values lower than 0.200 will be selected to the next step of the multivariable meta-regression analysis. The multivariable meta-regression was conducted using Protective Index as dependent variable and all others, which selected in the controlled univariate as independent variable. The multivariable model was conducted in a backwards approach, but in this case the exclusion was done manually in order to understand how the removal of no significant variable would affect the other variables. The statistical package used was the Stata software (Statacorp, Texas, USA).

This meta-regression approach allowed for attributing a given weight for each individual experiment based on their standard error. Therefore, a multiple meta-regression analysis was performed, including all parameters together, generating a meta-regression final model. Values of p<0.05 were considered statistically significant and was retained in the final model.

## Results

### Literature search and study characteristics

**Fig 1:**
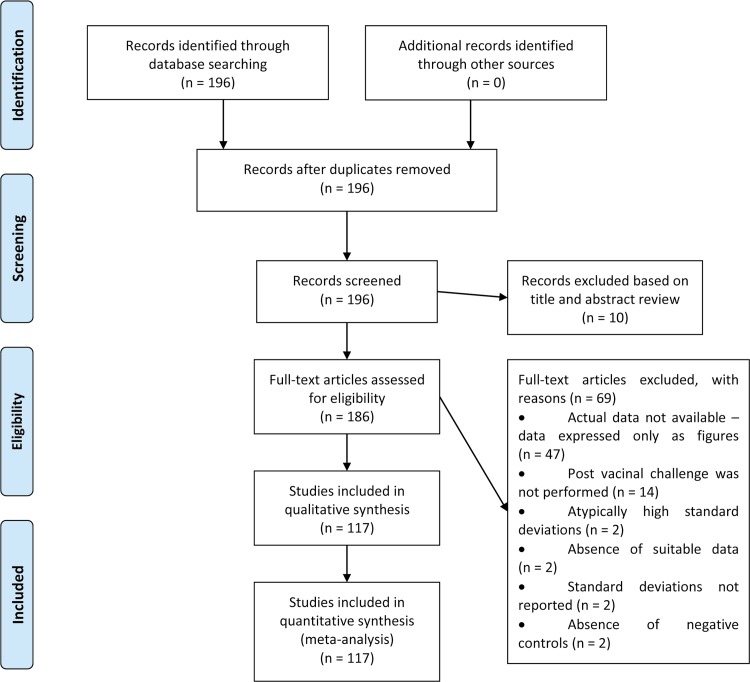
Flow chart describing the selection of articles for inclusion in the meta-analysis.

A total of 117 articles and data from 782 individual experiments were included in this study. Criteria for inclusion in this study are detailed in Fig 1. A total of 117 publications that met the criteria were selected for inclusion in this study [14, 17, 18, 20–28, 32, 33, 38, 40–141]. Therefore, a total of 782 individual experiments were analyzed. Raw data extracted from all 117 publications and each individual experiment are provided in the S2 Table.

### Protection against *Brucella* spp. induced by different categories of vaccines in mice—descriptive statistics

Currently, experimental subunit vaccines concentrate most of the research efforts in the field of *Brucella* vaccinology, since this category of vaccine accounted for 36.7%, followed by attenuated vaccine strains, which corresponded to 28.26% of all experiments. The others categories of experimental vaccines account for 13.04%, 8.69%, 8.43%, and 4.9%, in the case of mutant, DNA, inactivated, and vectored vaccines, respectively. Furthermore, the proteins that were more often used as subunit vaccines included: LPS fractions (n = 44), L7/L12 (n = 31), HS (n = 27), Omp19 (n = 22), Omp31 (n = 20), Omp16 (n = 17), Omp25 (n = 8), BLS (n = 8), SOD (n = 6), P39 (n = 6), BRF (n = 6), Omp28 (n = 5), and urease (n = 4).

**Fig 2:**
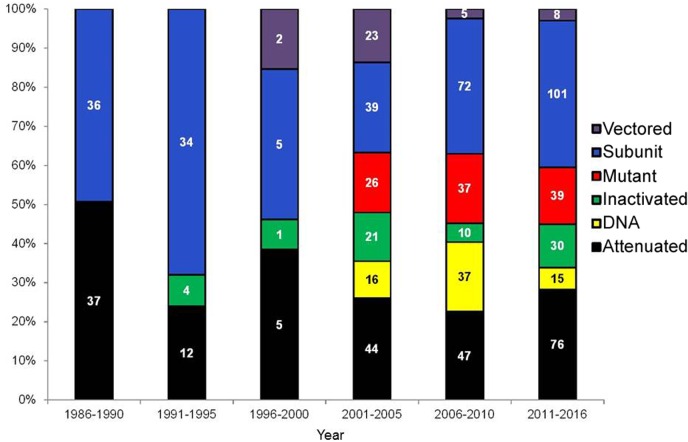
Time line with the number and percentage of experiments for brucellosis vaccine development according to the type of vaccine. Time intervals and corresponding number of experiments were: 1986–1990 (n = 73), 1991–1995 (n = 50), 1996–2000 (n = 13), 2001–2005 (n = 169), 2006–2010 (n = 208) e 2011–2016 (n = 269). The number of experiments for each data point is indicated in the graph.

Some categories of vaccines were established earlier while other types of vaccines emerged over the time of this study (1986–2016) as demonstrated in Fig 2. By the end of 1980’s (1986–1990) there were only experiments with attenuated and subunit vaccines. Inactivated vaccines appear between 1991 and 1995, whereas more diverse vaccine approaches have been developed and studied beginning in 2001. The period between 2011 and 2016 included the largest number of experiments (n = 269) when compared to the other intervals, which clearly indicates an increasing investment of research time and resources for brucellosis vaccine development.

**Fig 3:**
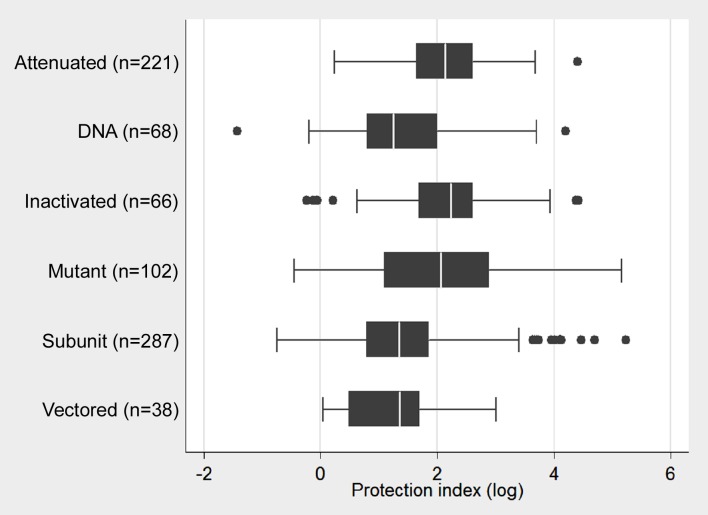
Protection index provided by different categories of experimental vaccine candidates against *Brucella* spp. infection. Values indicate the median, second and third quartiles (box), first and fourth quartiles (error bars). Outliers are indicated by dots. Median protection indexes were based on 782 independent experiments. The numbers of experimental groups per category are indicated between parentheses.

Data from 782 previously published experiments were grouped according to the category of experimental vaccines, namely naturally attenuated, mutant, inactivated, subunit, DNA, and vectored vaccines. Attenuated (n = 221), inactivated (n = 66) and mutant (n = 102) vaccines provided median protection index above 2, whereas subunit (n = 287), DNA (n = 68), and vectored (n = 38) vaccines provided protection indexes lower than 2 (Fig 3).

### Protection provided by experimental brucellosis vaccines over the past 30 years

**Fig 4:**
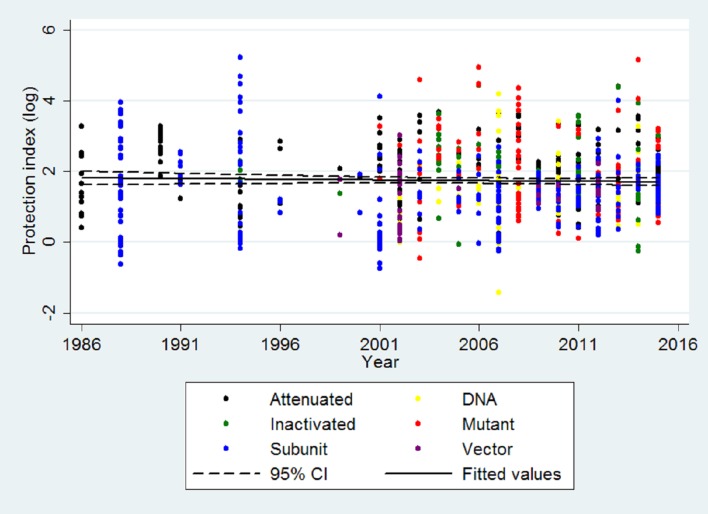
Linear regression of protection index over time for experimental vaccine candidates against *Brucella* spp. in the mouse model. All experimental vaccine categories (attenuated strains, n = 221; attenuated mutant strains, n = 102; inactivated vaccines, n = 66; subunit vaccines, n = 287; DNA vaccines, n = 68; and vectored vaccines, n = 38) were included in this analysis, corresponding to 782 individual experiments (r = -0.0038; r^2^ = 0.09%; p = 0.4052).

**Fig 5:**
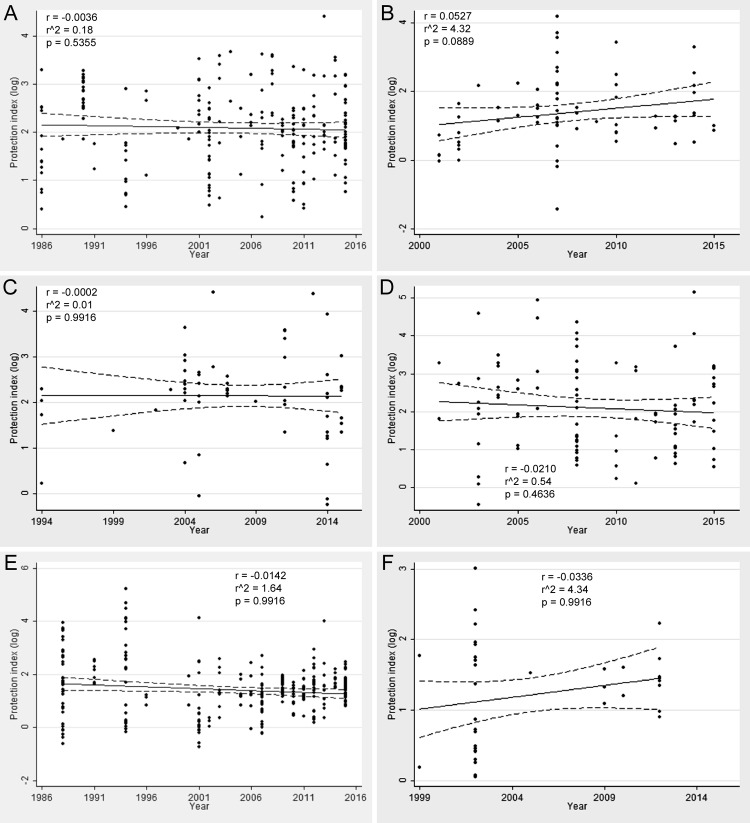
Linear regression of protection index over time of different categories of experimental vaccines against *Brucella* spp. in the mouse model. (A) attenuated strains (n = 221); (B) DNA vaccines (n = 68); (C) inactivated vaccines (n = 66); (D) attenuated mutant strains (n = 102); (E) subunit vaccines (n = 287); and (F) vectored vaccines (n = 38). Dots indicate each individual experiment, with solid trend lines and dotted lines indicating the confidence interval. Linear coefficients and p values are indicated in each graph.

In order to assess whether protection indexes have been improving over time, a correlation analysis was applied to protection indexes and the year of publication of each individual experiment over the past 30 years. When all categories of experimental vaccines are analyzed together, the trend line clearly demonstrates that there was no improvement of the protection indexes over the past 30 years, with a low negative and non significant linear coefficient (Fig 4). During this period of time, average protection indexes of experimental vaccines remained stable and close to 2 Log. A similar trend was observed when different vaccine categories were analyzed separately (Fig 5), with the exception of DNA vaccines that had a statistically significant positive correlation coefficient (Fig 5B). However, this trend to improving protection indexes over time in the case of DNA vaccines reflects the very low protection indexes of the early studies rather than high protection indexes since more recent studies have protection indexes that were in average below 2 Log (Fig 5).

### Parameters that influenced protection in the mouse model—descriptive statistics

A descriptive statistic analysis was performed considering the possible effect of several parameters, including mouse strain, vaccination routes, number of vaccinations, *Brucella* species used for experimental challenge, challenge route, and use of adjuvant, on protection indexes of experimental *Brucella* vaccines. Note this statistic descriptive does not take in account the weight of each experimental group, based in sample size and standard errors.

**Fig 6:**
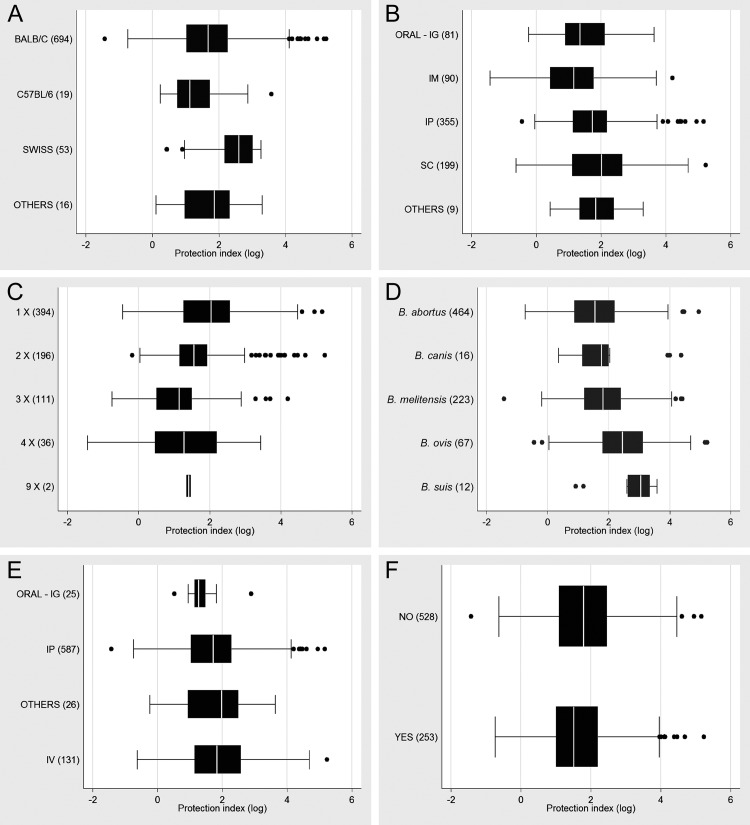
Protection indexes according to different parameters. All experimental vaccine categories were analyzed together and grouped according to: (A) the mouse strains used in each individual experiment; (B) vaccination route; (C) number of vaccinations; (D) the *Brucella* spp. species used for experimental challenge; (E) challenge route; and (F) use of adjuvant. The number of experimental groups for each parameter is indicated between parentheses. Values indicate the median, second and third quartiles (box), first and fourth quartiles (error bars). Outliers are indicated by dots.

Protection indexes were evaluated according to mouse strains, including Balb/c, Swiss, C57BL/6 and others, used in each one of the 782 experiments. In average, the highest levels of protection were observed in experiments using Swiss mice and its variations, including albino Swiss and outbreed Swiss CD-1 (Fig 6A). Balb/c is the most commonly used mouse strain for *Brucella* vaccine experiments, corresponding to 88.75% (694/782) of all experiments included in this study. In average, this strain provided lower protection indexes (1.7076), when compared to Swiss mice (2.3791) or other strains (1.7293), but higher than C57BL/6, which provided the lowest protection indexes (1.296) (Fig 6), when all vaccine categories were grouped together. Protection indexes provided by each mouse strain according to the category of vaccine (attenuated, DNA, inactivated, mutant, subunit, and vectored) are described in S1 Fig.

Different vaccination routes, i.e. oral and intragastic (ORAL/IG), intramuscular (IM), intraperitoneal (IP), subcutaneous (SC), and others (intranasal, intraesplenic, etc) provided similar protection indexes when all vaccine categories were analyzed together (Fig 6B). Protection indexes provided by different vaccination routes according to the vaccine category are detailed in S2 Fig.

The effect of the number of vaccinations, i.e. single vs. multiple vaccinations (2, 3, 4, and 9 vaccinations) on protection indexes were compared grouping all vaccine categories together. Interestingly, single vaccinations provided the highest median protection index (Fig 6C). Protection indexes provided by single or multiple vaccinations according to each vaccine category are described in S3 Fig.

Post vaccination challenges with different *Brucella* spp. species, namely *B*. *abortus*, *B*. *canis*, *B*. *melitensis*, *B*. *ovis*, and *B*. *suis*, were compared. A marked variation in protection indexes were observed against these virulent challenge species, with nearly two logs of difference in protection indexes between the lower and higher protection indexes, and challenge with *B*. *suis* resulted in the highest median protection index, when all vaccine categories were analyzed together (Fig 6D). Protection indexes provided by different vaccine categories against different *Brucella* spp. is described in S4 Fig.

The effect of the route of challenge on the protection index was also evaluated after analyzing all vaccine categories together. The median protection indexes obtained with challenge through different routes, i.e. oral and intragastric (ORAL–IG), intraperitoneal (IP), other (intranasal, intraesplenic, etc) e intravenous (IV), were quite similar (Fig 6E). Protection indexes provided by different routes of challenge according to each vaccine category are described in S5 Fig.

When analyzing all vaccine categories together, protection indexes provided by experimental vaccines with or without adjuvant were similar (Fig 6F). Importantly the use of adjuvant is largely restricted to some categories of experimental vaccines, as detailed in S6 Fig.

### Meta-analysis estimations

Random effects meta-analysis was conducted using 782 experimental groups from the 117 selected papers estimating the protraction index and testing for heterogeneity. This procedure was made for the experimental groups divided by type of vaccine as well. All estimations show high heterogeneity suggesting the necessity of use the meta-regression in order to access which factor is affecting the protection index. The results are displayed in the Table 1.

**Table 1:** Random effect meta-analysis results.

Type of vaccine	N	Estimation of PI	CI 95% of the estimation*		Heterogeneity test p-value
			Lower	Upper	
**All**	787	1.711	1.650	1.773	> 0.001
**Attenuated**	221	2.083	1.964	2.202	> 0.001
**DNA**	68	1.408	1.163	1.654	> 0.001
**Inactivated**	66	2.148	1.929	2.367	> 0.001
**Mutant**	102	2.052	1.831	2.274	> 0.001
**Subunit**	287	1.357	1.273	1.441	> 0.001
**Vector**	38	1.165	0.943	1.387	> 0.001

* CI: confidence interval, which indicates that under the same experimental conditions values would have that range in 95% of the time.

### Bivariate analyses

In order to select variables to be included in the multivariate meta-regression model, a bivariate meta-regression analysis was performed considering each of the variables controlled by vaccine category, i.e. a bivariate analysis (Table 2). Variables studied included: vaccine category, mouse strain, vaccination route, number of vaccinations, use or adjuvant, *Brucella* species used for challenge, challenge route, and interval between challenge and euthanasia. Naturally attenuated vaccine strains with an average protection index of 2.079 were significantly more protective (p<0.001) than DNA, subunit and vectored vaccines, which had average protection indexes of 1.377, 1.369, and 1.180, respectively. In contrast, protection indexes provided by inactivated and mutant vaccine strains (2.758 and 2.527, respectively) were statistically similar to that of the naturally attenuated vaccine strains.

**Table 2:** Bivariate meta-regression analysis of variables influencing the protection indexes of experimental brucellosis vaccines.

			Confidence interval***	
Variable	Coefficient*	p value**	Lower limit	Upper limit
**Vaccine category—Attenuated reference–Overall p-value < 0.001**				
**DNA**	-0.7025	<0.001*	-0.9597	-0.4453
**Inactivated**	0.0679	0.607	-0.1916	0.3275
**Mutant**	-0.04485	0.695	-0.2690	0.1793
**Subunit**	-0.7107	<0.001*	-0.8751	-0.5463
**Vector**	-0.8990	<0.001*	-1.2208	-0.5772
**Constant**	2.0799	<0.001*	1.9560	2.2038
**Mouse strain—Balb/c reference–Overall p-value < 0.001**				
**C57BL/6**	-0.6272	0.003*	-1.0439	-0.2104
**Swiss**	0.4197	0.002*	0.1555	0.6839
**Others**	-0.2984	0.203	-0.7583	0.1615
**Constant**	2.0589	<0.001*	1.9251	2.1927
**Route of vaccination–oral/intragastric reference–Overall p-value < 0.001**				
**Intramuscular**	-0.3571	0.081	-0.7591	0.0447
**Intraperitoneal**	0.2123	0.083	-0.0280	0.4526
**Subcutaneous**	0.4790	<0.001*	0.2226	0.7354
**Others**	0.4581	0.177	-0.2070	1.1233
**Constant**	1.7265	<0.001*	1.4697	1.9832
**Number of vaccinations–Single vaccination reference–Overall p-value < 0.001**				
**Two**	0.3872	0.002*	0.1453	0.6291
**Three**	-0.2241	0.108	-0.4974	0.0491
**Four**	-0.2645	0.197	-0.6667	0.1376
**Nine**	0.5179	0.437	-0.7895	1.8253
**Constant**	2.0597	<0.001*	1.9412	2.1783
**Adjuvant—without adjuvant reference**				
**Adjuvant use**	0.2937	0.002*	0.1111	0.4763
**Constant**	2.0665	<0.001*	1.9428	2.1901
**Species challenge—*B*. *abortus* reference–Overall p-value < 0.001**				
***B*. *canis***	0.4031	0.084	-0.054	0.8603
***B*. *melitensis***	0.1947	0.011*	0.0456	0.3438
***B*. *ovis***	0.8208	<0.001*	0.5739	1.0677
***B*. *suis***	0.8088	0.003*	0.2751	1.3425
**Constant**	1.9541	<0.001*	1.8232	2.085
**Challenge route–oral/intragastric reference–Overall p-value = 0.009**				
**Intraperitoneal**	0.1676	0.375	-0.2034	0.5386
**Others**	-0.4428	0.870	-0.5747	0.4861
**Intravenous**	0.4419	0.029*	0.4438	0.8395
**Constant**	1.8772	<0.001*	1.4957	2.2588
**Interval (days) between challenge and euthanasia**				
	-0.004	0.165	-0.0096	0.001
**Constant**	2.1665	<0.001*	1.9916	2.3414

* Positive regression coefficients indicate that the variable has higher protection indexes than the reference variable when statistically significant. Negative regression coefficients indicate the opposite.

** Statistically significant p values (p < 0.05).

*** Confidence interval indicates that under the same experimental conditions values would have that range in 95% of the time.

Evaluation of mouse strains considering Balb/c as the reference strain, with a protection index of 2.058, indicated that it had significantly higher protection indexes when compared to C57BL/6 (p = 0.003) that had a median protection index of 1.43. Conversely, Swiss mice had protection indexes (2.478) that were significantly higher than those of Balb/c mice (p = 0.002), whereas no significant differences were observed among “other” strains of mice and Balb/c (Table 2).

Meta-regression analysis of vaccination routes, considering the oral/intragastric route as reference, demonstrated that this route, with a protection index of 1.726, was significantly less protective (p<0.001) than the subcutaneous route (2.205). Protection indexes provided by intramuscular, intraperitoneal, and others (2.083, 1.938, and 2.184, respectively) were similar to the oral/intragastric route (Table 2).

Considering one single vaccination as reference with a protection index of 2.059, two vaccinations with a protection index of 2.446 provided better protection (p = 0.002) than single vaccinations. Conversely, three, four or nine vaccinations, with protection indexes of 1.835, 1.795, and 2.576, respectively, were statistically similar (p>0.05) to single vaccinations (Table 2).

The use of adjuvant resulted in a significantly better (p = 0.002) protective index (2.359), when compared to vaccination without adjuvant that resulted in a protective index of 2.066 (Table 2).

The analysis of challenge species, considering *B*. *abortus* as the reference with a protection index of 1.954 demonstrated that protection indexes against *B*. *melitensis*, *B*. *ovis*, and *B*. *suis* (2.148, 2.774, and 2.762, respectively) were significantly higher when compared to *B*. *abortus* (Table 2). Conversely, the protection index against *B*. *canis* (2.357) was similar to that of *B*. *abortus* (p<0.05).

Bivariate meta-regression analysis also considered the route of challenge, with the oral/intragastric route with a protection index of 1.877 as the reference. The intravenous (IV) route, with a protection index of 2.318, was significantly more protective than the reference. Protection indexes provided by the intraperitoneal (IP) or “other” routes (2.044 and 1.833, respectively) were statistically similar to the reference (Table 2).

Importantly, considering that vaccine experiments are not standardized, we evaluated the effect of the interval between challenge and measurement of the protective index, and the number of days between challenge and euthanasia of experimental animals did not significantly influenced the protective index (Table 2).

### Multivariate meta-regression

A meta-regression model was developed including all vaccine categories (attenuated, DNA, inactivated, mutant, subunit, and vectored) considering the protection index as a dependent variable and the other parameters (mouse strain, route of vaccination, number of vaccinations, use of adjuvant, challenge *Brucella* species) as independent variables (Table 3).

**Table 3:** Multivariate meta-regression analysis of variables influencing the protection indexes of experimental brucellosis vaccines.

		Standard		Confidence interval***	
Variable	Coefficient*	Error	p value**	Lower limit	Upper limit
**Vaccine category—Attenuated reference–Overall p-value < 0.001**					
**DNA**	-0.1826	0.2291	0.426	-0.6325	0.2672
**Inactivated**	-0.1333	0.139	0.338	-0.6325	0.1397
**Mutant**	-0.0145	0.1126	0.898	-0.2356	0.2066
**Subunit**	-1.0207	0.1453	<0.001*	-1.3059	-0.7355
**Vector**	-1.0774	0.1947	<0.001*	-1.4597	-0.695
**Mouse strain—Balb/c reference–Overall p-value = 0.020**					
**C57BL/6**	-0.4214	0.2086	0.044	-0.8309	-0.0119
**Swiss**	0.1386	0.1912	0.469	-0.2368	0.5141
**Others**	-0.5824	0.2524	0.021*	-1.0780	-0.0868
**Route of vaccination–oral/intragastric reference–Overall p-value = 0.007**					
**Intramuscular**	-0.4072	0.1929	0.035*	-0.7859	-0.0285
**Intraperitoneal**	-0.0454	0.1242	0.715	-0.2893	0.1985
**Subcutaneous**	0.1466	0.1316	0.266	-0.1118	0.4051
**Others**	0.3178	0.3136	0.311	-0.2979	0.9335
**Number of vaccinations—Single vaccination reference–Overall p-value < 0.001**					
**Two**	0.3943	0.1236	<0.001*	0.1516	0.6369
**Three**	-0.0459	0.1439	0.750	-0.3284	0.2366
**Four**	-0.2248	0.2096	0.284	-0.6363	0.1866
**Nine**	0.5015	0.644	0.436	-0.7629	1.766
**Adjuvant—without adjuvant reference**					
**Adjuvant use**	0.1745	0.0927	0.060	-0.0075	0.3565
**Species challenge—*B*. *abortus* reference–Overall p-value < 0.001**					
***B*. *canis***	0.157	0.2281	0.491	-0.2908	0.6048
***B*. *melitensis***	0.1652	0.0788	0.036*	0.0106	0.3198
***B*. *ovis***	0.7301	0.1287	<0.001*	0.4774	0.9828
***B*. *suis***	0.7793	0.3802	0.041*	0.0329	1.5257
**Constant**	1.8983	0.1349	<0.001*	1.6335	2.1632

* Positive regression coefficients indicate that the variable has higher protection indexes than the reference variable when statistically significant. Negative regression coefficients indicate the opposite.

** Statistically significant p values (p < 0.05).

*** Confidence interval indicates that under the same experimental conditions values would have that range in 95% of the time.

Subunit and vectored vaccines provided significantly lower protection indexes when compared to attenuated vaccines (p<0.001), which was considered the reference vaccine category. Protection indexes provided by DNA, inactivated or mutant vaccines were statistically similar (p<0.05) to the reference (Table 3).

Regarding the mouse strain used in the experiment, C57BL/6 and Swiss mice resulted in protection indexes that were statistically similar to the reference Balb/c strain (Table 3). Interestingly, “other” mouse strains, which included mouse strains that were knockout for genes related to immunity on a 129/Sv background, resulted in a lower protection index (p = 0.021) when compared to the reference (Table 3).

With the exception of the intramuscular route of vaccination that provided lower protection index when compared to the oral/intragastric (p = 0.035), the other vaccination routes (intraperitoneal, subcutaneous, and others) provided similar protection when compared to the reference (Table 3). Two vaccinations performed better than the reference that was one single vaccination (p<0.001), whereas three, four or nine vaccination did not improve the protection index when compared to the reference (Table 3).

Experimental vaccines provided significantly higher protection indexes against *B*. *melitensis*, *B*. *ovis*, and *B*. *suis* when compared to the reference challenge with *B*. *abortus* (Table 3), whereas the use of adjuvant did not have significant effect on the protection index (Table 3).

### Source of publications on brucellosis vaccinology

The data used in this study was obtained from 117 scientific articles, which were grouped according to the journal in which they were published. Frequencies of publications in different journals are detailed S3 Table.

## Discussion

Brucellosis remains as one of the most important zoonotic diseases in the world, which justifies the large number of studies aiming to develop new and improved vaccines [10]. A meta-analysis based on brucellosis vaccine development experiments in the mouse model was performed in this study. A temporal analysis indicates that protection indexes remained stable over the past 30 years, which may indicate that the knowledge accumulated during the past decades did not necessarily translated into better protection when considered the mouse as a model. Another way to interpret this unexpected and disturbing finding is that the mouse model may have a limited range of protection when it comes to brucellosis, which may have resulted in stable protection indexes over time. Limited knowledge on protective immune resposes of mice and natural host species of *Brucella* spp. may also be a factor limiting advancement of this field. Furthermore, traditional vaccine strategies, particularly those based on the use of attenuated strains [79,88,142] provided better protection when compared to new strategies such as subunit, DNA, and vectored vaccines. In the case of live attenuated vaccine strains there is a clear correlation between results obtained in the mouse model and actual protection in the preferred host species [10,11,143,144]. Indeed, vaccine strains such as *B*. *abortus* S19 and *B*. *melitensis* Rev.1 are known to generate a robust immune response [11,143], and to induce significant levels of protection against *B*. *abortus* in cattle and *B*. *melitensis* in sheep and goats [10,11,144].

The mouse has been largely used as an experimental model for *Brucella* spp. infection [36]. This model allows for calculating the protection index that is based on the difference between the number of CFU (in Log) in the spleens of non vaccinated controls and vaccinated mice [39]. Thus, a higher protection index indicates a better protection provided by a given experimental vaccine. Experimentally, the protection index is very important for *Brucella* sp. vaccinology, which contrasts to other pathogens that are lethal, for which protection may be assessed by prevention of lethality in the mouse model [145]. Importantly, correlation between protection index in the mouse model and protection in the preferred host species is not clear for most of the recently developed experimental vaccines. For instance, we have recently developed a *B*. *ovis* attenuated mutant vaccine candidate strain that lacks an ABC transporter [36], which influences the *virB*-encoded Type IV secretion system [146] thus interfering with intracellular trafficking [147]. This vaccine strain provided only moderate protection in the mouse model, yielding a protection index of approximately 1.0 [120], whereas it surprisingly provided a very strong protection against experimental challenge in rams, preventing shedding of the wild type strain in the semen and urine, accumulation of inflammatory cells in the semen, and gross or microscopic lesions induced by wild type *B*. *ovis*, resulting in sterile immunity under experimental conditions [148]. This lack of a direct correlation between protection in the mouse and the preferred host species may also be related to the fact that protection indexes varied according to the wild type *Brucella* species used for challenging, which may indicate that optimal levels of protection indexes may vary among different *Brucella* species.

This study demonstrated that attenuated live vaccine strains tend to provide higher levels of protection. Considering that *Brucella* spp. is an intracellular pathogen, attenuated vaccines tend to provide superior protection because the vaccine strain remains with the same tissue and cell tropism as the wild type strain, thus mimicking a natural infection [149]. In fact, *B*. *abortus* S19 and *B*. *melitensis* Rev1 are largely used as vaccine strains worldwide. Although these vaccine strains generate high levels of protection against disease, there are considerable drawbacks since they both have residual virulence for their hosts, they cause human infections and disease, and they interfere with routine serological assays since they generate a an antibody response against smooth *Brucella* lipopolysaccharide (LPS). Additionally, the Rev 1 vaccine strain is resistant to streptomycin, one of the antibiotics used for brucellosis treatment in human patients [11,76]. Conversely, the *B*. *abortus* RB51 vaccine strain provides protection against the disease in cattle [150], and it has the advantage of not interfering with the standard serological tests since this strain has a rough LPS [119], but this strain is resistant to rifampicin, which is used for brucellosis treatment in human patients [11]. Mouse experiments demonstrated that RB51 protects against experimental challenge with several *Brucella* spp. species, including *B*. *melitensis*, *B*. *ovis*, *B*. *abortus*, and *B*. *suis* [88]. Thus, *Brucella* mutant strains carrying a rough LPS have been used in several vaccine experiments [11,15]. However, mutant rough strains provide lower levels of protection when compared to smooth attenuated vaccines such as Rev 1 [74,151].

Beginning in 2000, a large number of experiments evaluated mutant attenuated *Brucella* strains as vaccine candidates. For the same reasons discussed concerning naturally attenuated strains, these mutant strains tend to provide protection in the mouse model. A limiting factor for these vaccines is the fact that some of these mutants have poor persistence in the host, which may not allow enough time for exposure of the vaccine strain to the immune system, thus preventing appropriate levels of protection [152–154]. However, delivery systems that promote a slow delivery of the vaccine strain may overcome this limitation [120,148]. The mutagenesis in these cases usually targets genes that are required for virulence or survival in the host [93,153,155,156]. Mutant whose deleted genes are required during the early stages of infection are quickly eliminated by the host immune system [153] so they tend to generate insufficient protective immunity [157,158]. There is a great interest in the generation of mutant strains that carry a rough LPS, such as RB51, since these strains do not interfere with the most commonly used serologic diagnostic methods [11,101]. However, rough strains tend to be rapidly eliminated from the host, which results in lower levels of protection [101].

This study demonstrated that, in general, subunit vaccines provided lower levels of protection, which may be due to limitations to identify the most protective antigens, but it is reasonable to hypothesize that one single antigen may not be sufficient to trigger a strong protective immune response against *Brucella* spp. [159,160,161].

In this study, some parameters affected protection against experimental challenge in the mouse model. Balb/c is the most commonly used mouse strain for *Brucella* vaccine experiments [16]. Importantly, protection indexes are influenced by the mouse strain. Indeed, although C57BL/6 and Swiss mice provided protection indexes that were similar to those of Balb/c, other strains, which included knockout strains for immune genes, provided lower protection indexes. With the exception of the intramuscular route of vaccination, all other vaccination routes provided similar levels of protection, including the subcutaneous route that is one of the preferable routes for practical purposes. The efficacy of the subcutaneous route of vaccination is in agreement with previous studies [16]. Another parameter that may influence protection, particularly in the case of subunit or DNA vaccines is the number of vaccinations, with two vaccination providing better results than single vaccination.

This study associated descriptive statistics with a meta-regression analysis, which is a powerful tool for advancing research on animal health [162]. A previous meta-analysis study on *Brucella* vaccinology have identified factors that may influence experimental outcomes in experiments evaluating whole organism vaccine formulations [16]. This study was more inclusive, covering most of the relevant *Brucella* vaccine research performed using the mouse model over the past three decades. The identification of variables that significantly influence protection indexes in the mouse model, clearly indicates that more standardized experimental protocols are urgently required to generate data that is more reproducible and with higher prediction value for vaccine performance in the preferred host species. Comparing with a previous meta-analysis study, which was restricted to whole organism vaccines [16], we found variables that are equally significant for other kinds of vaccines. For instance, vaccine category, mouse strain, vaccination route, challenge pathogen strain, challenge route, and challenge-killing interval, influenced protection in the previous study [16] as well as in this more comprehensive meta-analysis. Therefore, this study largely expands the knowledge previously gained with meta-analysis on *Brucella* vaccinology [16].

A critical aspect of the mouse model for *Brucella* vaccine development is the lack of standardized experimental conditions, which has been previously reviewed [163]. Although the mouse is a well established model for *Brucella* infection and vaccinology [36, 163], and in spite of very specific recommendations by the World Organisation for Animal Health (OIE) for employing the mouse as a model for predicting protective potential against brucellosis in ruminants [39], there is a wide range of parameters in experimental protocols, including sex, age and strain of mice, vaccination and challenge routes, time elapsed between vaccination and challenge and/or between challenge and assessment of splenic bacterial loads, among others. This fact makes comparisons between studies and laboratories very unreliable.

Potential limitations of this study may be associated with restrictions of the original database, although PubMed covers the vast majority of relevant papers on the field of experimental *Brucella* vaccinology. Absence of publication of negative results may also have influenced the outcome of this study, although similar levels of negative results would be expected among different categories of experimental vaccines.

## Conclusions

In conclusion, the importance of brucellosis as a threat for human health as well as due to economic losses for the animal industry [1,9], justifies the enormous scientific effort to develop better vaccines that lack residual pathogenic potential for animals and humans [19]. However, in spite of the large number of publication over the past 30 years, our results indicate that there is not clear trend to improve the protective potential of these experimental vaccines, which may at least in part explain why none of these new vaccine formulations or strategies has reached the market.

## Supporting Information

S1 Fig Protection index according to the mouse strain experimentally used for different categories of experimental vaccines against *Brucella* spp. infection. Vaccine categories (attenuated strains, DNA vaccines, inactivated vaccines, attenuated mutant strains, subunit vaccines, and vectored vaccines were regrouped according to the mouse strain experimentally used (Balb/c, C57BL/6, Swiss, and others). Attenuated vaccines: Balb/c, n = 166; C57BL/6, n = 9; Swiss, n = 34; others, n = 12. DNA vaccines: Balb/c, n = 67; C57BL/6, n = 1. Inactivated vaccines: Balb/c, n = 60; Swiss, n = 6. Mutant vaccines: Balb/c, n = 89; C57BL/6, n = 6; Swiss, n = 4; others, n = 3. Subunit vaccines: Balb/c, n = 274; C57BL/6, n = 3; Swiss, n = 9; others, n = 1. Vectored vaccines: Balb/c, n = 38. Values indicate the median, second and third quartiles (box), first and fourth quartiles (error bars). Outliers are indicated by dots.
(TIF)

S2 Fig Protection index according to the route of vaccination for different categories of *Brucella* spp. experimental vaccines. Vaccine categories (attenuated strains, DNA vaccines, inactivated vaccines, attenuated mutant strains, subunit vaccines, and vectored vaccines) were regrouped according to the route of vaccination (intragastric and oral, n = 81; intramuscular, n = 90; intraperitoneal, n = 355; subcutaneous, n = 199; others, n = 9). Attenuated vaccines: intragastric and oral, n = 12; intraperitoneal, n = 119; subcutaneous, n = 48. DNA vaccines: intramuscular, n = 62; others, n = 4. Inactivated vaccines: oral, n = 20; intraperitoneal, n = 19; subcutaneous, n = 26; others, n = 1. Mutant vaccines: oral, n = 5; intraperitoneal, n = 79; subcutaneous, n = 14. Subunit vaccines: oral, n = 25; intramuscular, n = 28; intraperitoneal, n = 119, others, n = 4. Vectored vaccines: oral, n = 19; intraperitoneal, n = 19. Values indicate the median, second and third quartiles (box), first and fourth quartiles (error bars). Outliers are indicated by dots.
(TIF)

S3 Fig Protection index according to the number of vaccinations for different categories of experimental vaccines against *Brucella* spp. Vaccine categories (attenuated strains, DNA vaccines, inactivated vaccines, attenuated mutant strains, subunit vaccines, and vectored vaccines) were regrouped according to the number of vaccinations (1x, n = 394; 2x, n = 196; 3x, n = 111; 4x, n = 36; 9x, n = 2). Attenuated vaccines: 1x, n = 211; 2x, n = 6; 4x, n = 1). DNA vaccines: 1x, n = 6; 2x, n = 2; 3x, n = 34; 4x, n = 26). Inactivated vaccines: 1x, n = 4; 2x, n = 13; 3x, n = 6; 4x, n = 3. Mutant vaccines: 1x, n = 97; 3x, n = 1. Subunit vaccines: 1x, n = 32; 2x, n = 148; 3x, n = 68; 4x, n = 3. Vectored vaccines: 1x, n = 4; 2x, n = 27; 3x, n = 2; 4x, n = 3; 9x, n = 2. Values indicate the median, second and third quartiles (box), first and fourth quartiles (error bars). Outliers are indicated by dots.
(TIF)

S4 Fig Protection indexes according to the challenge *Brucella* spp. species for different categories of experimental vaccines. Vaccine categories (attenuated strains, DNA vaccines, inactivated vaccines, attenuated mutant strains, subunit vaccines, and vectored vaccines) were regrouped according to the *Brucella* spp. species used for experimental challenge (*B*. *abortus*, *B*. *canis*, *B*. *melitensis*, *B*. *ovis*, and *B*. *suis*). Attenuated vaccines: *B*. *abortus*, n = 140; *B*. *melitensis*, n = 60; *B*. *ovis*, n = 12; *B*. *suis*, n = 9. DNA vaccines: *B*. *abortus*, n = 33; *B*. *canis*, n = 2; *B*. *melitensis*, n = 27; *B*. *ovis*, n = 6. Inactivated vaccines: *B*. *abortus*, n = 28; *B*. *canis*, n = 2; *B*. *melitensis*, n = 26; *B*. *ovis*, n = 7; *B*. *suis*, n = 3. Mutant vaccines: *B*. *abortus*, n = 40; *B*. *canis*, n = 4; *B*. *melitensis*, n = 47; *B*. *ovis*, n = 11. Subunit vaccines: *B*. *abortus*, n = 194; *B*. *canis*, n = 8; *B*. *melitensis*, n = 54; *B*. *ovis*, n = 31. Vectored vaccines: *B*. *abortus*, n = 29; *B*. *melitensis*, n = 9. Values indicate the median, second and third quartiles (box), first and fourth quartiles (error bars). Outliers are indicated by dots.
(TIF)

S5 Fig Protection index according to the challenge route for different experimental vaccine categories against *Brucella* spp. Vaccine categories (attenuated strains, DNA vaccines, inactivated vaccines, attenuated mutant strains, subunit vaccines, and vectored vaccines) were regrouped according to the route of challenge (oral and intragastric, n = 25; intraperitoneal, n = 587; others, n = 26; intravenous, n = 131). Attenuated vaccines: oral and intragastric, n = 5; intraperitoneal, n = 185; others, n = 4; intravenous, n = 23. DNA vaccines: oral and intragastric, n = 1; intraperitoneal, n = 48; intravenous, n = 15. Inactivated vaccines: intraperitoneal, n = 35; others, n = 14; intravenous, n = 17. Mutant vaccines: oral and intragastric, n = 1; intraperitoneal, n = 93; others, n = 4. Subunit vaccines: oral and intragastric, n = 16; intraperitoneal, n = 191; others, n = 4; intravenous, n = 75. Vectored vaccines: oral and intragastric, n = 2; intraperitoneal, n = 35; intravenous, n = 1. Values indicate the median, second and third quartiles (box), first and fourth quartiles (error bars). Outliers are indicated by dots.
(TIF)

S6 Fig Protection index according to the use of adjuvant for different categories of experimental vaccines against *Brucella* spp. Vaccine categories (attenuated strains, DNA vaccines, inactivated vaccines, attenuated mutant strains, subunit vaccines, and vectored vaccines) were regrouped according to the use or not of adjuvant (no, n = 528; yes, n = 253). Attenuated vaccines: no, n = 213; yes, n = 7. DNA vaccines: no, n = 61; yes, n = 7. Inactivated vaccines: no, n = 44; yes, n = 22. Mutant vaccines: no, n = 96; yes, n = 6. Subunit vaccines: no, n = 84; yes, n = 203. Vectored vaccines: no, n = 30; yes, n = 8. Values indicate the median, second and third quartiles (box), first and fourth quartiles (error bars). Outliers are indicated by dots.
(TIF)

S1 Table PRISMA Checklist. (DOC)

S2 Table Raw data extracted from all 117 publications and 782 individual experiments. (XLSX)

S3 Table Distribution of the 117 articles included in this study according to the journal they were published. (DOC)
