# Supplementary figures and images for: Meta-Analysis and Advancement of Brucellosis Vaccinology
Source: PLoS One. 2016 Nov 15;11(11):e0166582. doi: 10.1371/journal.pone.0166582 (PMC5112997; doi:10.1371/journal.pone.0166582)

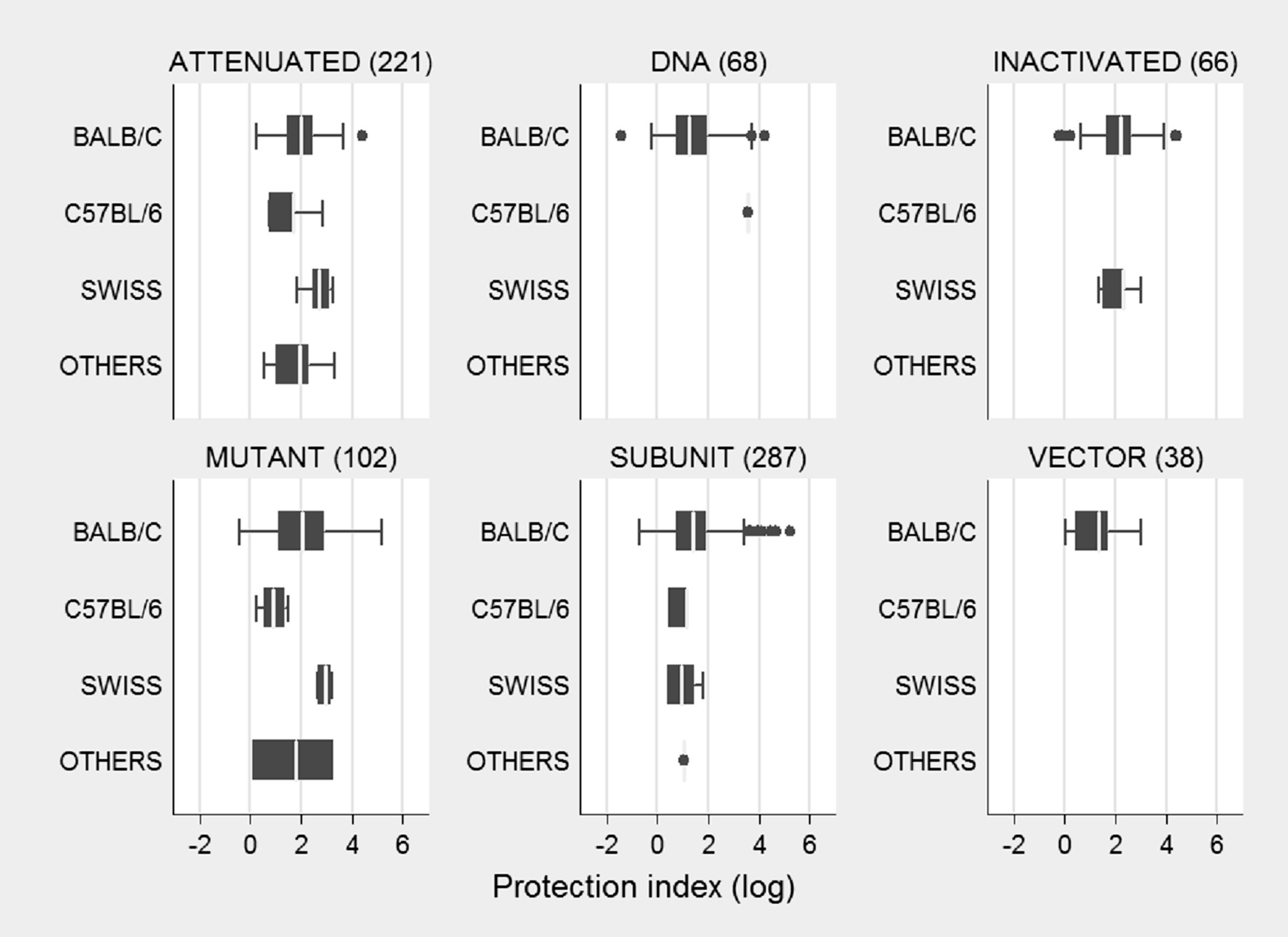

Supplement: S1 Fig — Vaccine categories (attenuated strains, DNA vaccines, inactivated vaccines, attenuated mutant strains, subunit vaccines, and vectored vaccines were regrouped according to the mouse strain experimentally used (Balb/c, C57BL/6, Swiss, and others). Attenuated vaccines: Balb/c, n = 166; C57BL/6, n = 9; Swiss, n = 34; others, n = 12. DNA vaccines: Balb/c, n = 67; C57BL/6, n = 1. Inactivated vaccines: Balb/c, n = 60; Swiss, n = 6. Mutant vaccines: Balb/c, n = 89; C57BL/6, n = 6; Swiss, n = 4; others, n = 3. Subunit vaccines: Balb/c, n = 274; C57BL/6, n = 3; Swiss, n = 9; others, n = 1. Vectored vaccines: Balb/c, n = 38. Values indicate the median, second and third quartiles (box), first and fourth quartiles (error bars). Outliers are indicated by dots. (TIF) [file pone.0166582.s001.tif]

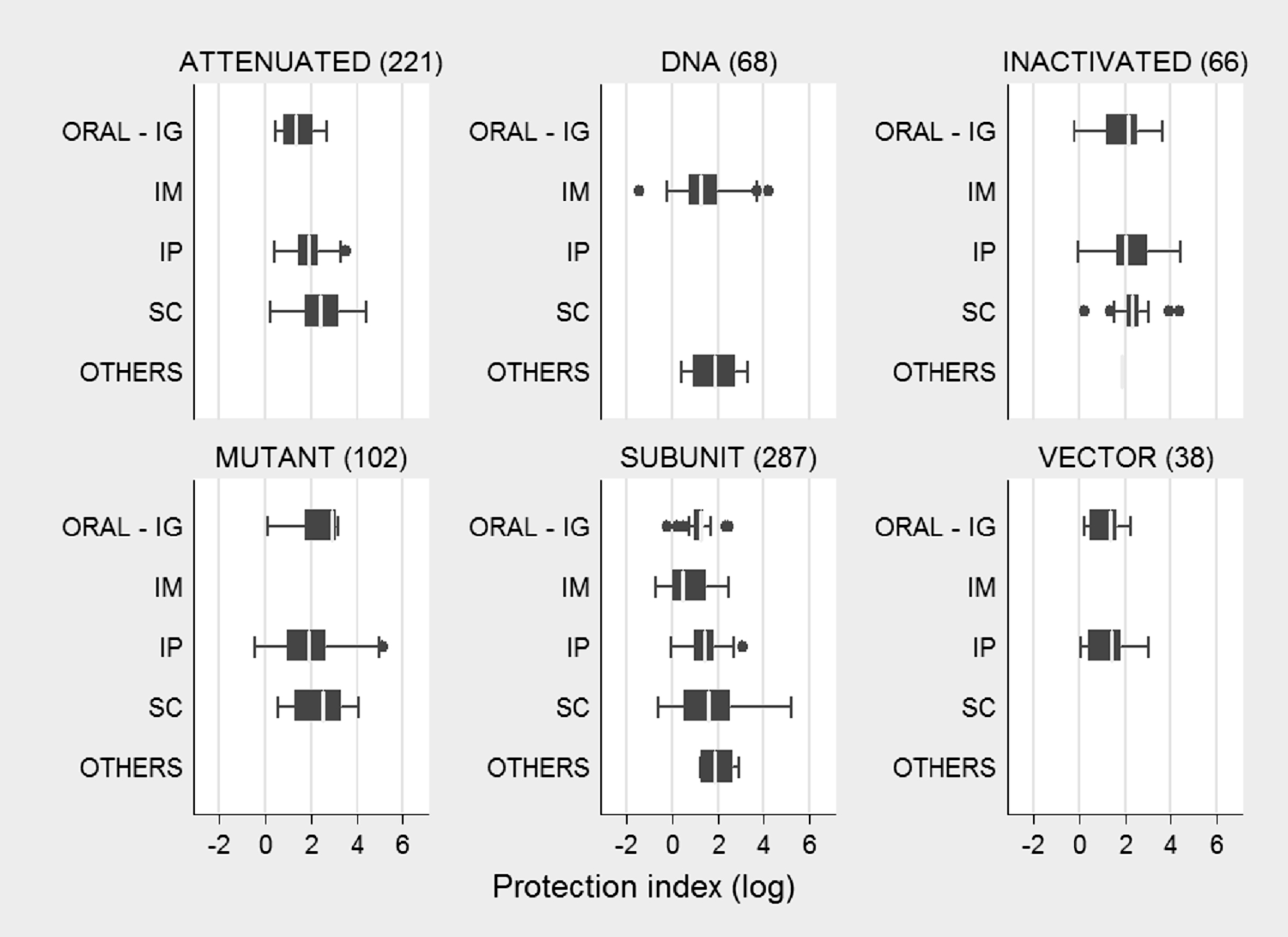

Supplement: S2 Fig — Vaccine categories (attenuated strains, DNA vaccines, inactivated vaccines, attenuated mutant strains, subunit vaccines, and vectored vaccines) were regrouped according to the route of vaccination (intragastric and oral, n = 81; intramuscular, n = 90; intraperitoneal, n = 355; subcutaneous, n = 199; others, n = 9). Attenuated vaccines: intragastric and oral, n = 12; intraperitoneal, n = 119; subcutaneous, n = 48. DNA vaccines: intramuscular, n = 62; others, n = 4. Inactivated vaccines: oral, n = 20; intraperitoneal, n = 19; subcutaneous, n = 26; others, n = 1. Mutant vaccines: oral, n = 5; intraperitoneal, n = 79; subcutaneous, n = 14. Subunit vaccines: oral, n = 25; intramuscular, n = 28; intraperitoneal, n = 119, others, n = 4. Vectored vaccines: oral, n = 19; intraperitoneal, n = 19. Values indicate the median, second and third quartiles (box), first and fourth quartiles (error bars). Outliers are indicated by dots. (TIF) [file pone.0166582.s002.tif]

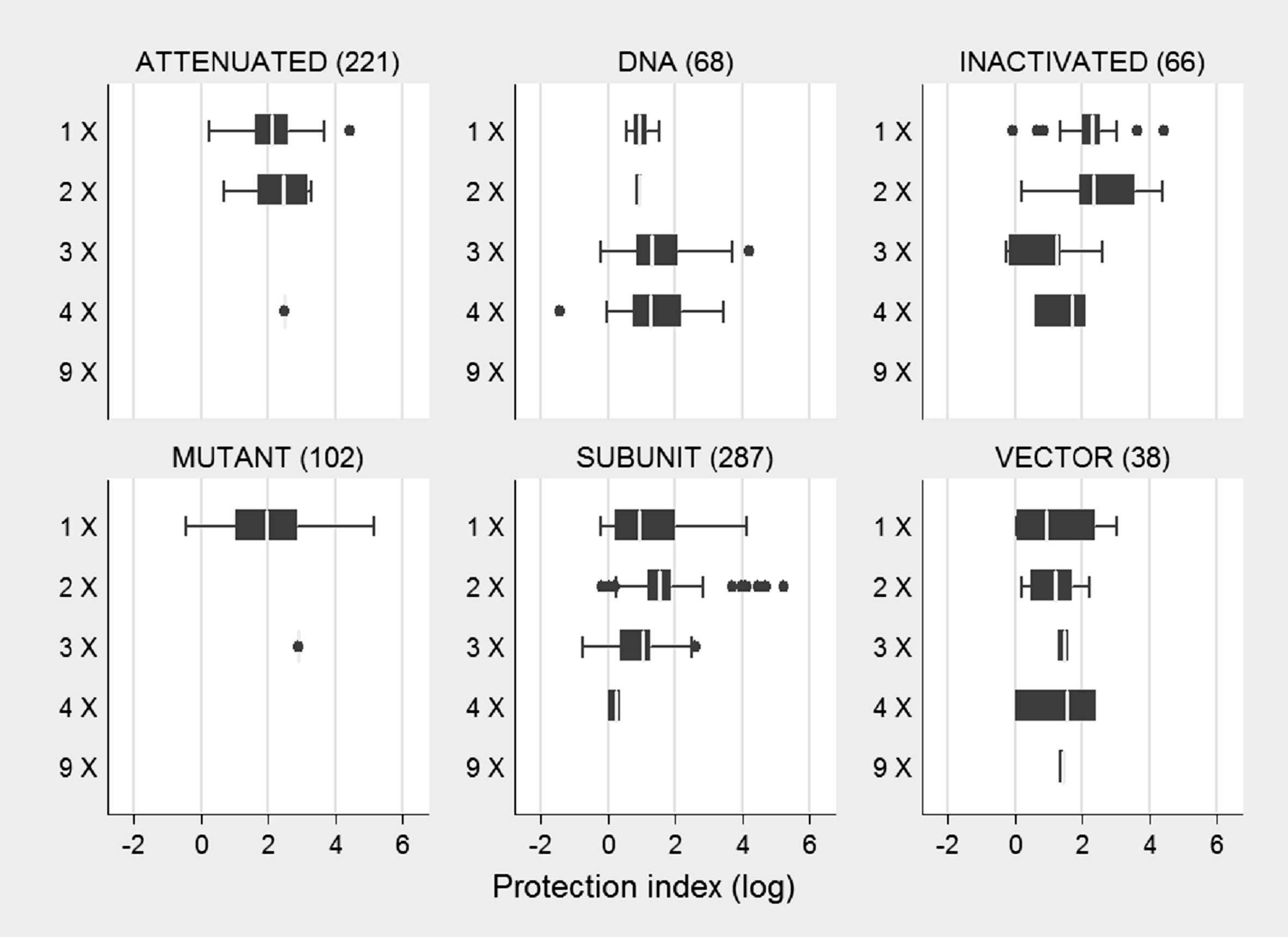

Supplement: S3 Fig — Vaccine categories (attenuated strains, DNA vaccines, inactivated vaccines, attenuated mutant strains, subunit vaccines, and vectored vaccines) were regrouped according to the number of vaccinations (1x, n = 394; 2x, n = 196; 3x, n = 111; 4x, n = 36; 9x, n = 2). Attenuated vaccines: 1x, n = 211; 2x, n = 6; 4x, n = 1). DNA vaccines: 1x, n = 6; 2x, n = 2; 3x, n = 34; 4x, n = 26). Inactivated vaccines: 1x, n = 4; 2x, n = 13; 3x, n = 6; 4x, n = 3. Mutant vaccines: 1x, n = 97; 3x, n = 1. Subunit vaccines: 1x, n = 32; 2x, n = 148; 3x, n = 68; 4x, n = 3. Vectored vaccines: 1x, n = 4; 2x, n = 27; 3x, n = 2; 4x, n = 3; 9x, n = 2. Values indicate the median, second and third quartiles (box), first and fourth quartiles (error bars). Outliers are indicated by dots. (TIF) [file pone.0166582.s003.tif]

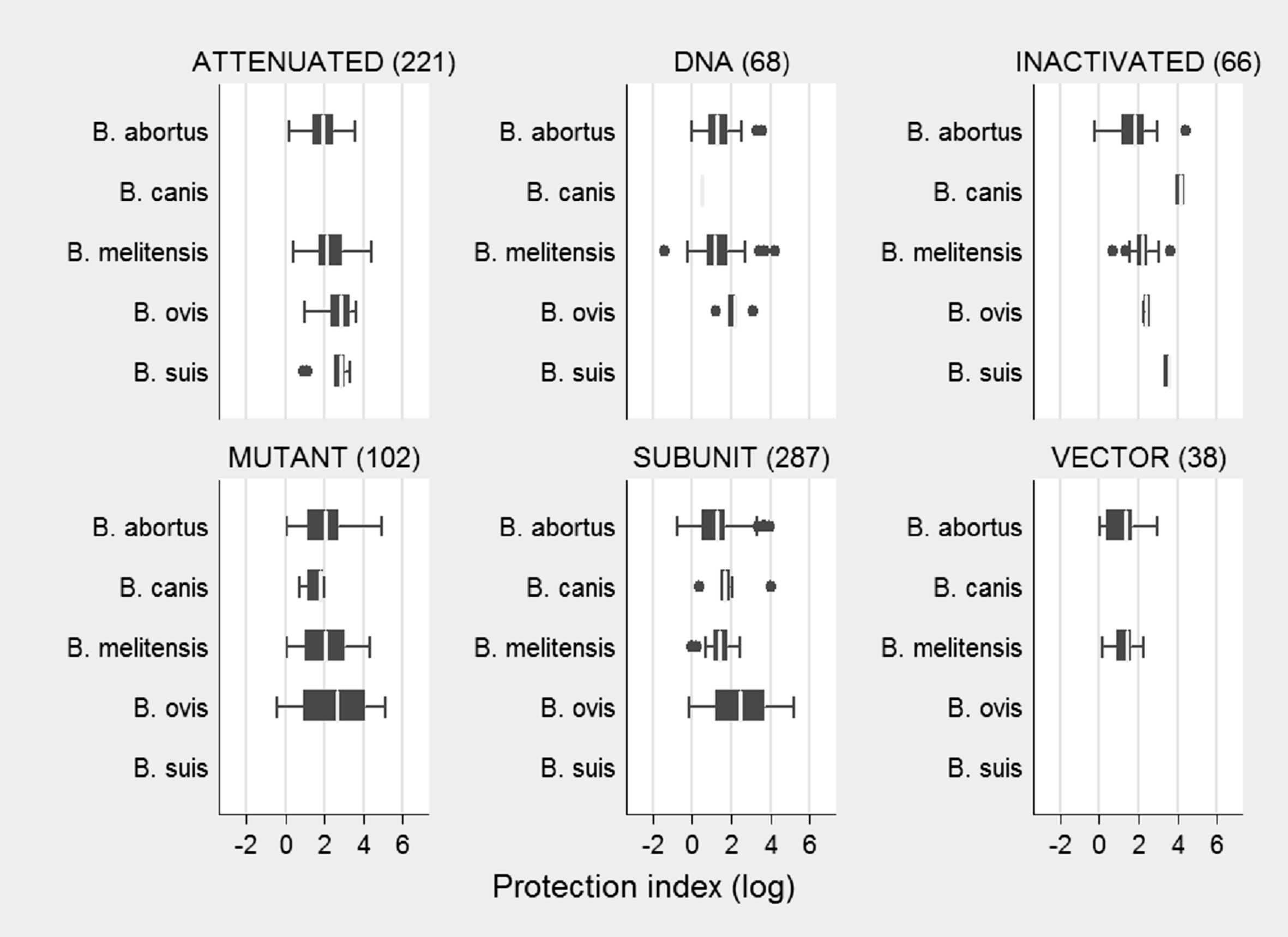

Supplement: S4 Fig — Vaccine categories (attenuated strains, DNA vaccines, inactivated vaccines, attenuated mutant strains, subunit vaccines, and vectored vaccines) were regrouped according to the Brucella spp. species used for experimental challenge (B. abortus, B. canis, B. melitensis, B. ovis, and B. suis). Attenuated vaccines: B. abortus, n = 140; B. melitensis, n = 60; B. ovis, n = 12; B. suis, n = 9. DNA vaccines: B. abortus, n = 33; B. canis, n = 2; B. melitensis, n = 27; B. ovis, n = 6. Inactivated vaccines: B. abortus, n = 28; B. canis, n = 2; B. melitensis, n = 26; B. ovis, n = 7; B. suis, n = 3. Mutant vaccines: B. abortus, n = 40; B. canis, n = 4; B. melitensis, n = 47; B. ovis, n = 11. Subunit vaccines: B. abortus, n = 194; B. canis, n = 8; B. melitensis, n = 54; B. ovis, n = 31. Vectored vaccines: B. abortus, n = 29; B. melitensis, n = 9. Values indicate the median, second and third quartiles (box), first and fourth quartiles (error bars). Outliers are indicated by dots. (TIF) [file pone.0166582.s004.tif]

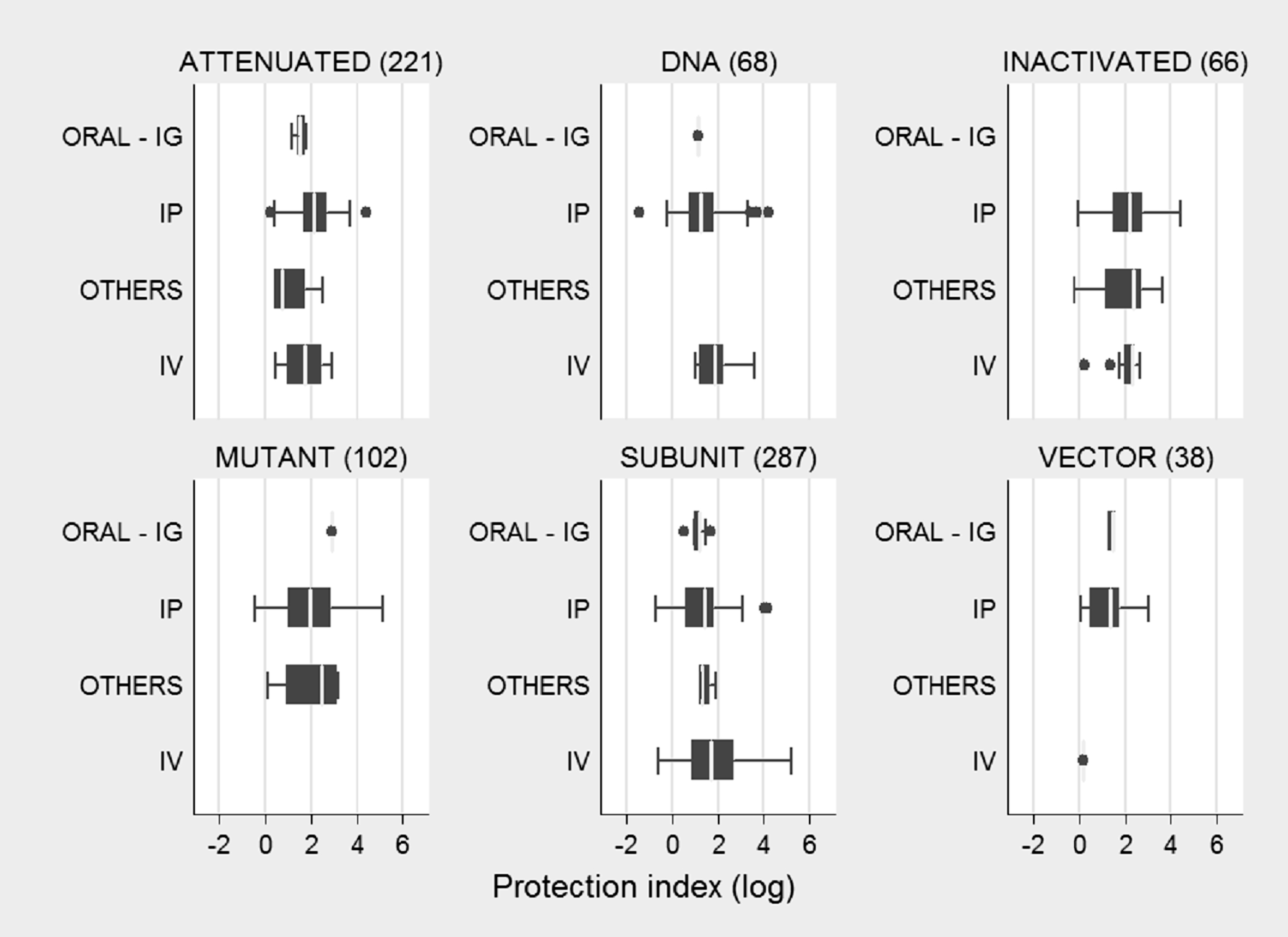

Supplement: S5 Fig — Vaccine categories (attenuated strains, DNA vaccines, inactivated vaccines, attenuated mutant strains, subunit vaccines, and vectored vaccines) were regrouped according to the route of challenge (oral and intragastric, n = 25; intraperitoneal, n = 587; others, n = 26; intravenous, n = 131). Attenuated vaccines: oral and intragastric, n = 5; intraperitoneal, n = 185; others, n = 4; intravenous, n = 23. DNA vaccines: oral and intragastric, n = 1; intraperitoneal, n = 48; intravenous, n = 15. Inactivated vaccines: intraperitoneal, n = 35; others, n = 14; intravenous, n = 17. Mutant vaccines: oral and intragastric, n = 1; intraperitoneal, n = 93; others, n = 4. Subunit vaccines: oral and intragastric, n = 16; intraperitoneal, n = 191; others, n = 4; intravenous, n = 75. Vectored vaccines: oral and intragastric, n = 2; intraperitoneal, n = 35; intravenous, n = 1. Values indicate the median, second and third quartiles (box), first and fourth quartiles (error bars). Outliers are indicated by dots. (TIF) [file pone.0166582.s005.tif]

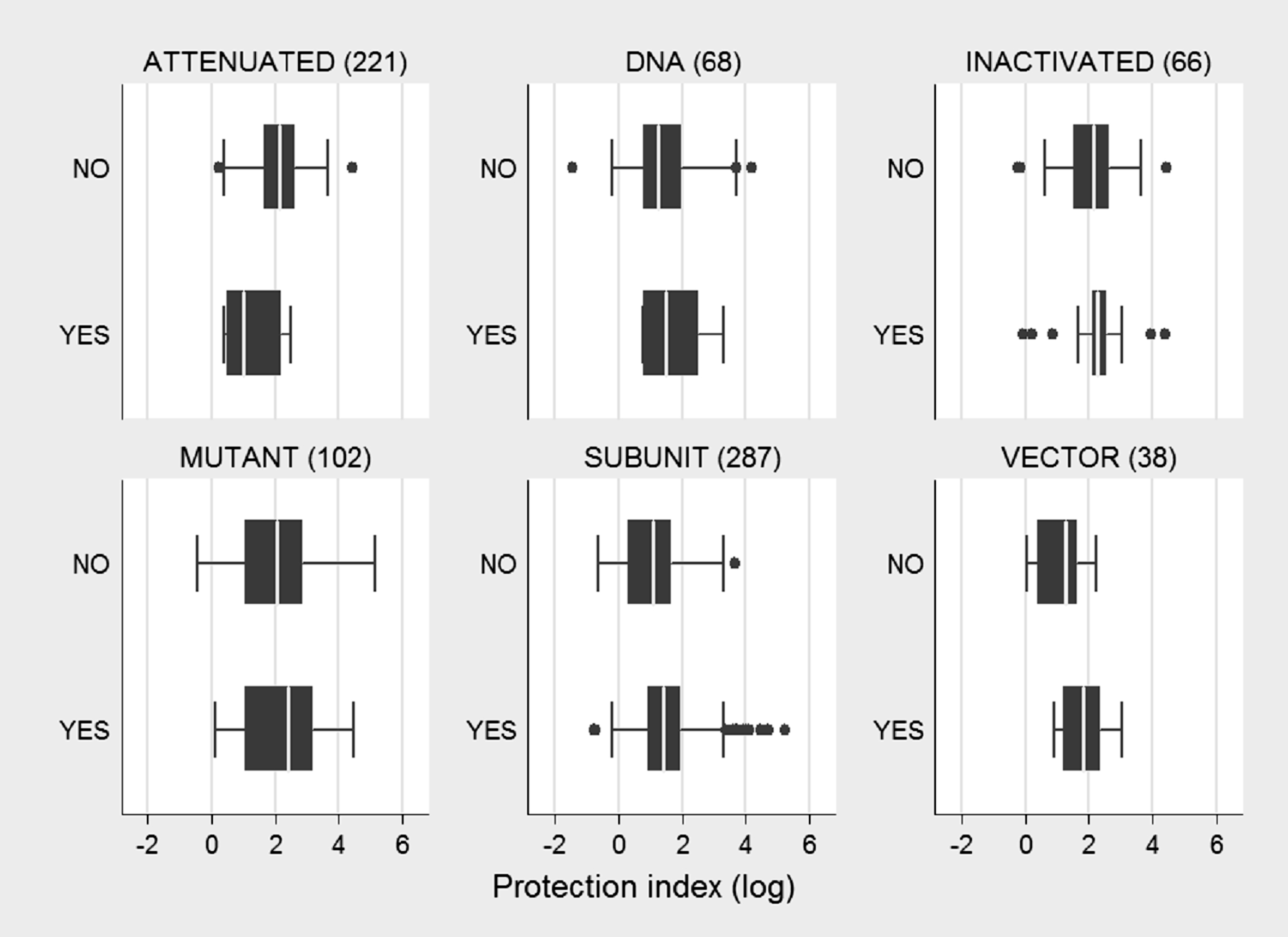

Supplement: S6 Fig — Vaccine categories (attenuated strains, DNA vaccines, inactivated vaccines, attenuated mutant strains, subunit vaccines, and vectored vaccines) were regrouped according to the use or not of adjuvant (no, n = 528; yes, n = 253). Attenuated vaccines: no, n = 213; yes, n = 7. DNA vaccines: no, n = 61; yes, n = 7. Inactivated vaccines: no, n = 44; yes, n = 22. Mutant vaccines: no, n = 96; yes, n = 6. Subunit vaccines: no, n = 84; yes, n = 203. Vectored vaccines: no, n = 30; yes, n = 8. Values indicate the median, second and third quartiles (box), first and fourth quartiles (error bars). Outliers are indicated by dots. (TIF) [file pone.0166582.s006.tif]
