# Supplementary material for: Meta-Analysis and Advancement of Brucellosis Vaccinology
Source: PLoS One. 2016 Nov 15;11(11):e0166582. doi: 10.1371/journal.pone.0166582 (PMC5112997; doi:10.1371/journal.pone.0166582)
Supplement: S3 Table — (DOC) [file pone.0166582.s009.doc]

**Supplementary Table 3**

Distribution of the 117 articles included in this study according to the journal they were published.

| **Journal** | **Frequency** | **Percent (%)** |
| --- | --- | --- |
| Vaccine | 35 | 29.91 |
| Infection and Immunity | 27 | 23.07 |
| Molecular Immunology | 7 | 5.98 |
| Clinical and Vaccine Immunology | 7 | 5.98 |
| Plos One | 4 | 3.41 |
| Veterinary Microbiology | 4 | 3.42 |
| Journal of Medical Microbiology | 3 | 2.56 |
| Veterinary Research | 2 | 1.71 |
| [World Journal of Microbiology and Biotechnology](http://www.springer.com/chemistry/biotechnology/journal/11274) | 2 | 1.71 |
| Biologicals | 2 | 1.71 |
| FEMS Immunology and Medical Microbiology | 2 | 1.71 |
| Immunobiology | 2 | 1.71 |
| Journal of Veterinary Science | 2 | 1.71 |
| Microbes and Infection | 2 | 1.71 |
| Veterinary Research Communications | 2 | 1.71 |
| APMIS | 1 | 0.85 |
| Archivos de Medicina Veterinária | 1 | 0.85 |
| Clinical and Diagnostic Laboratory Immunology | 1 | 0.85 |
| DNA and Cell Biology | 1 | 0.85 |
| Frontiers in Cellular and Infection Microbiology | 1 | 0.85 |
| Frontiers in Microbiology | 1 | 0.85 |
| Genetic Vaccines and Therapy | 1 | 0.85 |
| The Journal of Infection in Developing Countries | 1 | 0.85 |
| Journal of Controlled Release | 1 | 0.85 |
| Journal of Microbiology | 1 | 0.85 |
| Microbiology and Immunology | 1 | 0.85 |
| Research in Veterinary Science | 1 | 0.85 |
| The Journal of Immunology | 1 | 0.85 |
| Veterinary Immunology and Immunopathology | 1 | 0.85 |
| **Total** | **117** | **100** |
